# Combining Free Text and Structured Electronic Medical Record Entries to Detect Acute Respiratory Infections

**DOI:** 10.1371/journal.pone.0013377

**Published:** 2010-10-14

**Authors:** Sylvain DeLisle, Brett South, Jill A. Anthony, Ericka Kalp, Adi Gundlapallli, Frank C. Curriero, Greg E. Glass, Matthew Samore, Trish M. Perl

**Affiliations:** 1 Veterans Affairs Maryland Health Care System, Baltimore, Maryland, United States of America; 2 University of Maryland, Baltimore, Maryland, United States of America; 3 Veterans Affairs Salt Lake City Health Care System, Salt Lake City, Utah, United States of America; 4 University of Utah, Salt Lake City, Utah, United States of America; 5 Johns Hopkins University, Bloomberg School of Public Health, Baltimore, Maryland, United States of America; 6 Johns Hopkins Medical Institutions, Baltimore, Maryland, United States of America; St. Petersburg Pasteur Institute, Russian Federation

## Abstract

**Background:**

The electronic medical record (EMR) contains a rich source of information that could be harnessed for epidemic surveillance. We asked if structured EMR data could be coupled with computerized processing of free-text clinical entries to enhance detection of acute respiratory infections (ARI).

**Methodology:**

A manual review of EMR records related to 15,377 outpatient visits uncovered 280 reference cases of ARI. We used logistic regression with backward elimination to determine which among candidate structured EMR parameters (diagnostic codes, vital signs and orders for tests, imaging and medications) contributed to the detection of those reference cases. We also developed a computerized free-text search to identify clinical notes documenting at least two non-negated ARI symptoms. We then used heuristics to build case-detection algorithms that best combined the retained structured EMR parameters with the results of the text analysis.

**Principal Findings:**

An adjusted grouping of diagnostic codes identified reference ARI patients with a sensitivity of 79%, a specificity of 96% and a positive predictive value (PPV) of 32%. Of the 21 additional structured clinical parameters considered, two contributed significantly to ARI detection: new prescriptions for cough remedies and elevations in body temperature to at least 38°C. Together with the diagnostic codes, these parameters increased detection sensitivity to 87%, but specificity and PPV declined to 95% and 25%, respectively. Adding text analysis increased sensitivity to 99%, but PPV dropped further to 14%. Algorithms that required satisfying both a query of structured EMR parameters as well as text analysis disclosed PPVs of 52–68% and retained sensitivities of 69–73%.

**Conclusion:**

Structured EMR parameters and free-text analyses can be combined into algorithms that can detect ARI cases with new levels of sensitivity or precision. These results highlight potential paths by which repurposed EMR information could facilitate the discovery of epidemics before they cause mass casualties.

## Introduction

As the outbreak of severe acute respiratory syndrome (SARS)[1] and the continued threat of novel influenza strains that could produce pandemics illustrate [2], [3], human populations remain vulnerable to epidemics of acute respiratory infections (ARI). These outbreaks must be discovered as soon as possible if control measures are to be implemented in time to curb morbidity, mortality and socioeconomic disruptions [4].

The increasing availability of electronic health data offers the opportunity to enhance public health surveillance for infectious diseases, a process that has historically relied on slow and incomplete paper-based reporting [5]. Early electronic surveillance efforts have collected codified diagnoses, chief complaints, school absenteeism, over-the-counter medication sales and other independent data streams to detect the early stages of infectious illnesses, when mild signs and symptoms may not alarm clinicians [6], [7], [8], [9], [10], [11]. While the utility of these pre-diagnostic, so-called “syndromic” surveillance systems (SSS) has thus far been less than persuasive, there is now increasing evidence that the electronic transfer of diagnostic test results can lead to more complete and timely reporting of notifiable diseases to the public health authorities [12], [13], [14]. These results encourage the continued search for new approaches aimed at improving the timeliness and usefulness of surveillance alerts.

By assembling a rich array of clinical data around individual patients, a surveillance system rooted in the electronic medical record (EMR) could provide early and nuanced illness detection compared to current-generation SSS. Notwithstanding the challenges of protecting patient privacy, an EMR-based surveillance system could also support the efficient flow of information necessary to promptly investigate alerts and manage evolving epidemics [15], [16], [17]. To gain insight into the potential of the EMR as a pre-diagnostic surveillance tool, we asked how information automatically retrieved from a comprehensive EMR could be arranged to detect patients with symptoms suggestive of ARI. More specifically, we hypothesized that structured and free-text clinical entries into the Veterans Administration's (VA) comprehensive EMR could be automatically extracted, transformed and combined to improve ARI detection compared to the sole use of ICD-9 diagnostic codes, a common basic avenue to electronic disease detection.

## Results

### Study Population

The Maryland (n = 5,127) and the Utah (n = 10,250) study samples were representative of their respective outpatient populations and included mostly male veterans. They differed in racial composition: the Utah population was more than 90% Caucasian, whereas almost half of the Maryland veterans were African American (Table 1).

**Table 1:** Patients demographics.

		Maryland Site			Utah Site		
Category	Sub-category	Patient Base n = 206,561	Study Sample n = 5,127	ARI Cases n = 142	Patient Base n = 47,257	Study Sample n = 10,250	ARI Cases n = 138
Age	<21 y	0.2	0	0	0.3	0	0
	21–30 y	2.3	1.7	3.5	4.5	1.1	3.6
	31–40 y	7.9	4.7	11.3	5.5	2.7	3.6
	41–50 y	15.9	14.9	28.2	9.1	7.5	13
	51–60 y	20.6	24.4	25.4	23.3	23.7	37
	61–70 y	13.6	16.5	14.1	18.8	21.5	18.1
	71–80 y	20.7	25.9	12.7	23.1	30.5	15.2
	81–90 y	15.2	11.4	4.9	14.9	12.7	8.7
	91–100 y	2.4	0.5	0	0.5	0.2	0.7
Sex	Male	86	91	88.7	92	96.1	92
	Female	14	9	11.3	8	3.9	8
Race	White	56.2	52.3	49.5	93.4	93.8	90.1
	African	42.8	46.4	50.5	1.6	1.1	0
	Hispanic	0.5	0.7	0	3.4	3.5	4.6
	Other	0.5	0.6	0	1.5	1.5	5.3

Numbers within column 3-8 represent the percentage of the base, study sample and ARI populations.

### ARI Cases

Our review of the 15,377 sampled records identified a total of 280 ARI cases. ARI incidence during the 6-month study period was lower at the UT site (1.3%) than at the MD site (2.8%). On average, ARI cases were younger than the study population at both sites, with patients over the age of 70 representing 46% of the study sample but only 21% of ARI cases (Table 1). The distribution of ARI cases across outpatient or telephone care areas is shown in Table 2. Two-thirds of ARI cases were seen in the emergency room or in the same-day/walk-in primary care areas, where they made up ∼8% of the visits. Comparatively, ARI cases amounted to less than 1% of visits to routine care areas.

**Table 2:** Location of care of ARI patients.

Location of Care	% of Total Visits	% of Total ARI Cases	% of Visits with ARI
Emergency Room	12.9	58.6	8.3
Same-Day Appointments	2.1	8.6	7.5
Telephone Care	11.2	13.2	2.1
Routine (Primary Care)	63.7	18.6	0.5
Routine (Specialty Care)	9.3	1.1	0.2

Cough was the most commonly documented symptom of ARI (88% of cases), followed by fever/chills/night sweats (58%) and sore throat (45%). Eight (8) percent of ARI patients were febrile at the time of the encounter. Table 3 displays the frequency with which these and other less common symptoms and signs occurred, either alone or in selected combinations. Note that gastrointestinal symptoms were common complaints, even though they were not part of the ARI case definition.

**Table 3:** Symptoms and signs in ARI patients.

Symptoms/Signs		Cough	Fever or Chills or Night Sweats	Sore throat
	% of Total ARI Cases	% of Cases with the Additional Symptom		
**Cough**	88.2		57	42
**Fever or Chills or Night Sweats**	58.2	86		26
**Sore throat**	44.6	82	34	
**Myalgia or Arthralgia**	22.9	81	59	38
**Headache**	19.6	73	38	42
**Chest Pain-Pleuritic**	8.2	91	48	22
Gastrointestinal*	16.4	91	67	35
**Shortness of Breath**	12.9	92	81	17
**Malaise/Fatigue**	10.7	90	87	20
**Chest Pain-Non Pleuritic**	5.4	93	60	27
**Lymph node enlargement**	2.1	0	67	67
Neurological**	1.8	80	40	40
**Skin Lesion or Rash**	0.7	50	50	100

*: includes nausea, vomiting, diarrhea or abdominal pain.

**: includes neck stiffness, changes in mental status, photophobia, diplopia or other visual changes.

Percent of ARI patient population (n = 280, *column 2*) with selected symptoms or signs (*column 1*). Columns 3-5 represent the percent of ARI patients with the symptoms/signs shown in Column 1 who *also* have cough (*column 3*), fever/chills/night sweats (*column 4*) or sore throat (*column 5*).

### ARI CDAs That Use Structured EMR Parameters

**Figure 1:**
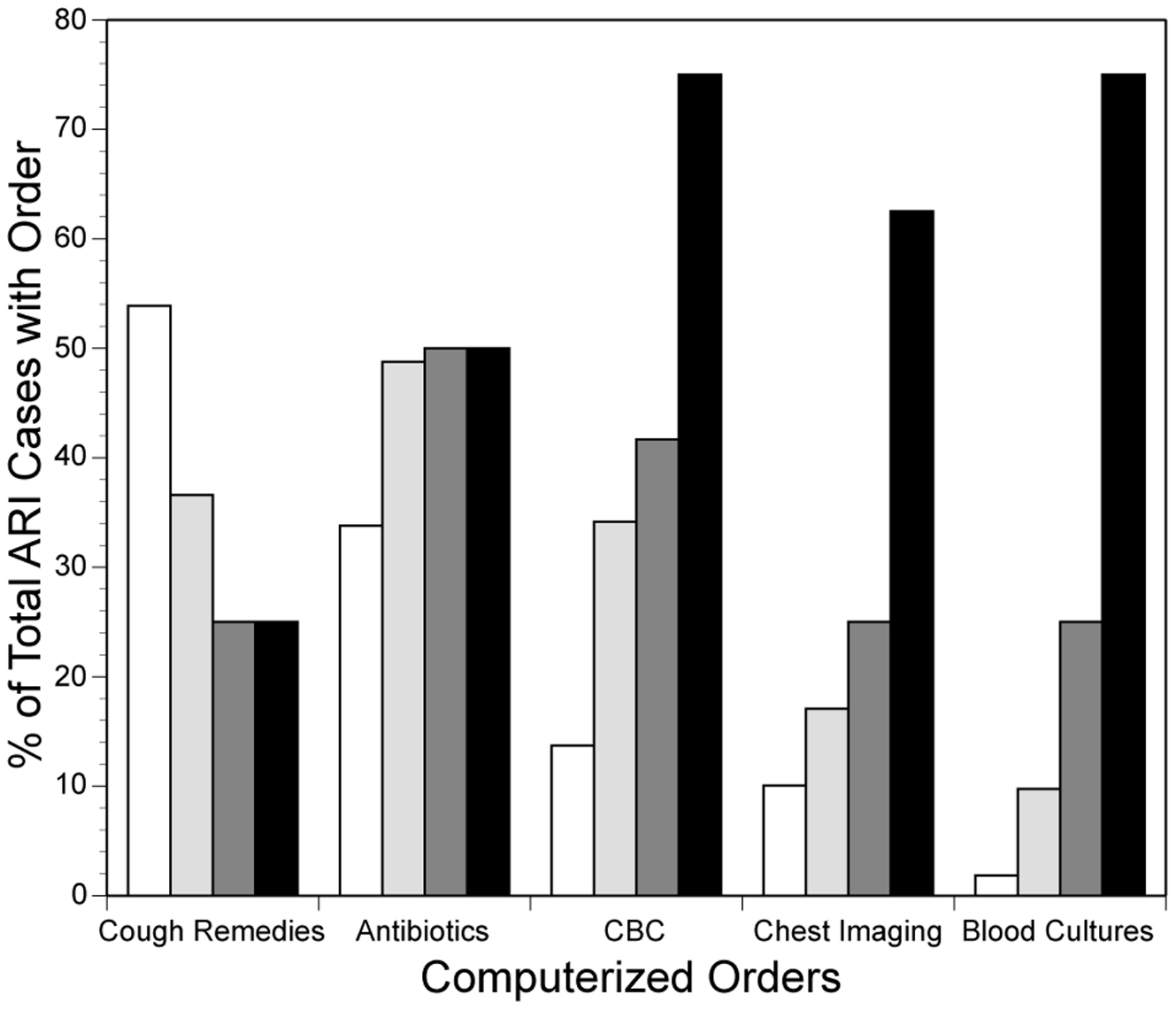
Relationship between medical orders and the number of abnormal vital signs. Frequency of ordering selected medications and tests (y axis) in ARI patients with normal vital signs (*white bar*), or who have an increasing number of abnormal vital signs (one (*light grey bar*), two (*dark grey bar*) or three (*black bar*). For each vital sign, “abnormal” was defined as follows: temperature ≥38°C, respiratory rate ≥22 breath per minute, heart rate ≥100 beats per minute.

Six of the EMR parameters considered were either not found or present in only one ARI patient, and did not contribute to ARI detection at either site. These included orders of diagnostic tests for influenza or for other respiratory tract pathogens, prescriptions for antivirals, antidiarrheals or antiemetics, and orders for sinus x-rays. The overall frequency at which other EMR parameters occurred at both sites is shown in Table 4. Providers wrote prescriptions aimed at relieving cough or cold symptoms in 65% of ARI patients, and blood tests or imaging in 27%. Rates at which CBC, chest imaging and blood cultures were obtained were low (Table 4) but increased with the number of abnormal vital signs (Fig. 1).

**Table 4:** Incidence of orders and abnormal vital signs in ARI patients.

Category	Subcategory	% of ARI Cases
Prescriptions	Cough Remedies	42.9
	Other Cold Remedies	17.5
	Antibiotics	33.8
Test Orders	CBC/Diff	13.7
	Chest Imaging	10
	Gram Stain	1.8
	Blood Culture	1.8
Vital Signs Abnormalities	Heart Rate ≥100 beats/min	13.6
	Respiratory Rate ≥22 breaths/min	10
	Temperature ≥38°C	8.2

Percent of ARI patients for who selected medications or tests were ordered, or who had abnormal vital signs. CBC/Diff stands for complete blood count with differential white blood cell count.

CDAs that did not include ICD-9 codes achieved a peak sensitivity of 80% and specificity of 69%, and were not retained (data not shown). Table 5 reports the composition and performance of illustrative CDAs that used ICD-9 codes either alone (CDAs 1-3) or a combination with other structured EMR parameters (CDA 4-9). ICD-9-only CDAs used by the BioSense (CDA 1) or the ESSENCE (CDA 2) SSS during our study period could be improved by adding three ARI-compatible ICD-9 codes (acute maxillary sinusitis (461.0), acute sinusitis NOS (461.9), and bronchitis NOS (490)) to the ESSENCE code set. The resulting ICD-9 code set, labeled “VA” (CDA 3), was used as a starting point for further CDA development. Five EMR parameters could improve ARI case detection when added to the VA ICD-9 codes: orders for cough remedies, CBC, gram stains of respiratory secretions, blood cultures and a temperature ≥38°C. Of these, the “Temperature ≥38°C” and the “Cough Remedies” parameters were significant at both study sites, whether used alone (CDAs 4 and 6) or together (CDA 8). In contrast, the blood culture parameter alone (CDA 5) or the addition of CBC and gram stains to other parameters (e.g. CDA 9) were significant at one site, but not at the other.

**Table 5:** Detection performance of CDAs that target ARI.

		Case-Detection Algorithm Number								
Category	Subcategory	1	2	3	4	5	6	7	8	9
CDA Component	BioSense ICD-9 Codes	•								
	Essence ICD-9 Codes		•							
	VA ICD-9 Codes			•	•	•	•	•	•	•
	OR Temperature ≥38°C				•				•	•
	OR Blood Culture					•		•		
	OR Cough Remedies						•	•	•	•
	OR CBC/Diff									•
	OR Gram stain									•
Maryland Site	Sensitivity	61	68	78	81	81	84	87	87	87
	Specificity	94	97	96	96	95	95	94	94	93
	Positive Predictive Value	23	37	36	35	33	31	30	30	27
	Area under the ROC	78	83	87	89	88	90	91	91	90
Utah Site	Sensitivity	64	74	80	80	80	85	85	86	87
	Specificity	91	98	97	97	97	96	95	95	92
	Positive Predictive Value	9	29	27	25	25	20	20	19	13
	Area under the ROC	78	86	89	89	89	91	90	91	90

Composition (*black dot* indicates that the component is included in the CDA) of ARI CDAs that combine parameters derived from structured EMR entries. Statistical performance can be compared at each study site.

### ARI CDAs That Use Free-Text EMR Entries

The Text-only CDA was as sensitive as a CDA that best combined ICD-9 codes with other structured clinical parameters (Table 6, compare CDA 10 to CDA 8). The “VA ICD-9 codes”, “Cough Remedies” and “Text” CDA components could be coupled through an OR operand to bring sensitivities up from 88% (CDA 10) to 97–99% (CDAs 11 and 12). While this maximized the estimated area under the ROC curve, gains in sensitivity were accompanied by losses in specificity (from 93 to 89%, CDA 10 to CDA 12). Because of the low incidence of ARI in this broad outpatient population (an average of 1.8% during our 6-month study period), these losses in specificity halved the positive predictive value (PPV) that had originally been obtained with CDA that used ICD-9 codes alone (Table 6, compare CDA 3 to CDA 12). We could design CDAs with high PPV (47–60%) using only structured EMR parameters, for example by requiring that both the “ICD-9 codes” and the “Temperature ≥38°C” parameters be satisfied (CDA 13). However, the resulting CDAs exhibited low sensitivities (e.g. 6%, CDA 13, and 38%, CDA 14). CDAs that had to satisfy both a query of structured EMR parameters as well as the text analysis exhibited similarly high PPVs (52–54%), but retained much higher sensitivities (69%, CDA 15, and 73%, CDA 16).

**Table 6:** Contribution of free-text analysis to ARI detection.

		Case-Detection Algorithm Number										
Category	Subcategory	3	4	6	8	10*	11	12	13	14	15	16
CDA Component	(VA ICD-9 Codes	•	•	•	•		•	•	•	•	•	•
	OR Temperature ≥38°C		•		•							
	OR Cough Remedies			•	•			•				•
	Text-Only; OR Text)					•	•	•				
	AND Temperature >38°C								•			
	AND Cough Remedies									•		
	AND Text										•	•
Performance	Sensitivity	79	80.5	84.5	86.5	88*	97	99	6	38	69	73
	Specificity	96.5	96.5	95.5	94.5	93	90	89	99.9	99.9	99.9	99.9
	Positive Predictive Value	31.5	30	25.5	24.5	18*	16	14	60	47	54	52
	Area under the ROC	88	89	90	90	90	94	94	53	68	84	86

*Performance of text-mining routines is usually reported using the term “Recall” instead of “Sensitivity” and “Precision” instead of “Positive Predictive Value”. Another common text-mining metric, the F-measure (2 * Precision * Recall/(Precision + Recall) is 29.9 for CDA #10.

Composition (*black dot* indicates that the component is included in the CDA) of ARI CDAs that combine parameters derived from structured and free-text EMR entries. Statistical performance was combined for both study sites. CDAs 3-12 seek increasing sensitivity; CDA 13-16 seek increasing PPV.

### Targeting ARI Patients With an Influenza-Like Illness (ILI)

The Centers of Disease Control and Prevention (CDC) defines an influenza-like illness (ILI) as an acute febrile illness (temperature of ≥37.8°C) accompanied by a cough and/or a sore throat. Thus defined, patients with ILI represent a subset of our broad ARI population. A Text-only CDA achieved a higher sensitivity for ILI than its ICD-9-only counterpart (Table 7, 92%, CDA 18 vs. 79%, CDA 17). Despite specificities of 91-96%, both detection approaches yielded PPVs in the 2–3% range. As could be expected, restricting cases retrieved through ICD-9 codes or text analysis to those patients who also have a temperature of at least 37.8°C increased the PPV more than 10-fold (CDAs 19 and 20) without decreasing sensitivity. Selecting febrile patients who also satisfied the [ICD-9-OR-Text] clause (CDA 21) identified all ILI cases and yet maintained a PPV of 34%. Alternatively, requiring patients to simultaneously satisfy the ICD-9 codes, text and temperature parameters maximized the PPV (68%) while keeping sensitivity above 70% (CDA 22).

**Table 7:** Performance of EMR-based CDA that target influenza-like illness.

		CDA Number					
Category	Subcategory	17	18	19	20	21	22
CDA Component	(VA ICD-9 Codes	•		•		•	•
	Text-Only; OR Text)		•		•	•	
	AND Text						•
	AND Temperature >37.8°C			•	•	•	•
Performance	Sensitivity	79	92	75	92	100	71
	Specificity	95.5	91	99.8	99.7	99.7	99.9
	Positive Predictive Value	2.7	2	36.7	34	34	68
	Area under the ROC	87	91	87	96	100	85

Composition (*black dot* indicates that the component is included in the CDA) of ILI CDAs that combine parameters derived from structured and free-text EMR entries. Statistical performance was combined for both study sites.

## Discussion

In this work, we combined selected structured EMR entries with a computerized analysis of free-text documentation to detect reference patients with acute respiratory infections with sensitivities up to 99%, or PPV up to 68%. The results support our working hypothesis that judicious use of information contained in the EMR can improve early disease detection compared to the sole use of diagnostic codes, and raise the possibility that harnessing the EMR as a data source may improve the overall performance of automated outbreak detection systems.

Case definitions used for influenza surveillance vary widely [18], reflecting both the variability of influenza's clinical presentation and differing views on what distinguishes an influenza-like illness from other ARI [19], [20]. Absent a standard, we crafted a case definition that would identify a broad population of patients with ARI. Most (78%) of our ARI patients had normal vital signs and thus probably suffered from mild, uncomplicated ARI [21]. Nevertheless, two-third of them felt ill enough to visit urgent or same-day care areas. Thus, the majority of patients who satisfied our case definition were seeking health services despite a mostly mild illness. These patients lie at the crosshair of syndromic surveillance.

We used a manual abstraction of respiratory symptoms from the EMR as a reference standard to uncover cases of ARI. This case-finding method may have underestimated the absolute incidence of ARI encounters, as busy providers may not document symptoms that are impertinent to a focused outpatient visit. Nevertheless, the abstraction, which was done on a large random sample of records, relied on information committed to a real-world EMR and thus represents a realistic benchmark against which to compare the performance of ARI detection algorithms relative to each other.

### Contribution of Diagnostic Codes

The reference review allowed an evaluation of a stalwart component of SSS, ICD-9 diagnostic codes. Our data support the suggestion that an early step toward system enhancement is to scrutinize the ICD-9 codes used to categorize cases [22]: additions of only a few codes improved both detection sensitivity and specificity. While our observed level of performance may not be duplicated in health systems where diagnostic codes are assigned by non-providers and/or have reimbursement repercussions, our findings reinforce results obtained in adult and pediatric emergency room populations [23], [24]. Note that the ICD-9 code parameter was retained statistically in all of the most successful ARI and ILI detectors, suggesting that diagnostic codes are not made redundant with the availability of a broad array of structured EMR parameters. Our results therefore suggest that ICD-9 codes represent a valuable, perhaps indispensible component of automated ARI detection algorithms, and bolster their widespread use.

### Contribution of Structured EMR Parameters Other Than Diagnostic Codes

Some of the non-ICD-9 parameters considered in this study had previously emerged as potentially useful for ARI surveillance. In particular, sales of selected over-the-counter or prescription medications [25], [26], [27], [28], [29], [30], [31], [32] and requests for diagnostic tests for influenza or for other respiratory pathogens [12], [33] have been shown to correlate with the seasonality of influenza and respiratory syncytial virus infections. Our study revisits similar parameters, adds new ones not yet formally investigated, but evaluates them in a different way i.e. for the accuracy with which they can detect reference ARI cases not already found by alternative parameters. This evaluation was made possible because EMR-derived parameters were not utilized as independent data streams. Rather, EMR parameters were related to each other at the single-patient level.

Amongst a broad field of candidate structured EMR entries, we found two that could complement ICD-9 codes to detect patients with ARI: new prescriptions directed at the symptom of cough and an elevation in measured body temperature recorded in an EMR field dedicated to vital signs. With 88% of ARI patients experiencing a new onset of cough, it is not surprising that prescriptions for cough remedies uncovered new ARI cases. While these results lend further support to the use of medications-related data elements for biosurveillance [29], [30], [31], the practical difficulty is to devise database queries that, over time, will continue to reflect the concept of “cough remedies”. Cough suppressants belong to a variety of drug classes (opioids, non-opioid antitussives, decongestants, antihistamines) that are often variably combined with each other and with other agents to relieve flu-like symptoms. Efforts to standardize competing drug terminologies and to develop methods to automatically update medication groupings [27], [28] must be encouraged if symptom-relief medications are to be routinely incorporated into surveillance systems.

Though significant statistically, the added contribution of the non-ICD-9 structured EMR parameters to ARI detection was admittedly modest. We attribute this low marginal utility at least in part to the mildness of the disease in the majority of our ARI patients. To wit, providers did not order any blood tests or chest imaging in 73% of these encounters. Our efforts at devising CDAs that target the subset of ARI patients with ILI hint at how fruitful incorporating structured non-ICD-9 EMR parameters may become as disease severity increases. For example, a parsimonious ILI CDA (VA ICD-9 codes AND Temperature >37.8°C, CDA 19) increased PPV by an order of magnitude with little losses in sensitivity compared to its ICD-9 codes-only counterpart. While we acknowledge that an ICD-9 code set optimized for ILI could narrow this performance gap, our observations that more diagnostic tests are ordered with increasing vital signs abnormalities (Fig. 1) raise the prospect that as disease severity increases, so will the value of the structured EMR data.

In contrast to the potential utility of the “cough remedies” and the “Temperature” parameters, specific tests and therapies targeting viral ARI were not utilized, and thus did not contribute detection value. Perhaps the simplest explanation for why providers didn't order tests for viral respiratory pathogens or anti-influenza drugs is that neither were felt to offer a meaningful therapeutic impact in non-hospitalized patients during our study period. Because these practice patterns could change quickly with the emergence or fear of a more severe illness, and for the important situational and logistical insight they may provide during an epidemic, we would certainly retain disease-specific diagnostic and therapeutic EMR entries in ARI CDAs meant for an operational surveillance system.

### Contribution Of Text Analysis

For surveillance purposes, text analyses have been applied to limited data sources within the EMR, including “chief complaint” fields [34], [35], [36], [37], [38], laboratory or imaging reports [34], [35], [38], [39], [40] or dictated hospital discharge summaries [41], [42]. We are aware of only one published study where ARI surveillance was performed by applying text analysis to the full outpatient notes typed in by the providers themselves [43]. This study illustrated the feasibility of using the full narrative text for surveillance in the ambulatory setting, and showed a temporal correlation between total daily ILI counts obtained using text analysis, queries of structured EMR data, categories of chief complaints or influenza isolates. Our work extends this knowledge in two significant ways. First, our reference review allowed us to provide insight into the performance of free-text mining per se. Despite ongoing challenges with non-standard grammar and abbreviations, spelling mistakes, lack of punctuation, copy-and-pasting, personal templates and checklists, our simple approach to free-text analysis discovered ARI cases with a sensitivity (88%) and a specificity (93%, CDA 10) not dissimilar to what could be reached with structured parameters (e.g. CDA 8). Second, the relational nature of EMR data allowed us to observe the extent to which free-text and structured data sources could complement each other for case detection. On the one hand, we found that combining the results of the two mining approaches through an “OR” logical operand yielded near-perfect sensitivity, albeit at the cost of a much reduced PPV. These results support the common-sense belief that, at least with a disease characterized by relatively mild symptoms, significant information resides exclusively in the provider's note. On the other hand, we found that combining the two approaches through an “AND” logical operand could increase PPVs well above 50% while retaining sensitivities in the 70% range (CDAs 15, 16, 22). These results suggest that cross-validating information obtained from structured data with that obtained from clinical narrative can reduce false-positive findings from either data sources and improve the noise environment through which a surveillance system must operate to uncover disease clusters. Whether or not the achievable gains in signal-to-noise ratio will outweigh the losses in detection sensitivity to improve the overall ability of the surveillance system to detect disease outbreaks will have to be determined empirically.

### Limitations

Several factors, some of which have already been mentioned, may limit the generalizability of our results: 1) factors related to the performance of our study at the VA health care system: a) the veterans study population is mostly male and excludes the pediatric population, a key target for ARI surveillance [44]; b) veterans health care utilization may differ from that observed in uninsured or privately insured individuals; c) clinical practices, documentation and coding habits by VA practitioners may differ from those observed in solo or group practices or in health systems subject to different financial or quality-control incentives; 2) factors related to our study period: optimal CDAs could differ outside the respiratory infection season, or during periods of heightened apprehension for an influenza epidemic; 3) factors related to our iterative CDA development process, which may have over adapted CDAs to VA's particular EMR implementation and to our sample dataset in particular, this despite our efforts to maintain a separation between development and validation data subsets; 4) factors related to our text mining approach: a) we did not employ a spell checker prior to applying the NegEx algorithm. Instead, we added common misspellings of negation and search terms (e.g. “deneis” instead of “denies”, “cocuphing” instead of “coughing”) identified during the chart review process. Thus, unencountered misspellings are left out of our final UMLS concept table and negation list; b) our adaptation of the NegEx algorithm is specific to the types of EMR note from only two VA medical centers. Further adaptations could therefore be needed for text documents extracted from the VISTA systems at other VA facilities.

### Summary

Our results confirms that it is feasible to harness the EMR for biosurveillance purposes and that judicious parameter selection coupled with free-text analyses can yield case-detection tools with performance characteristics not previously available through diagnostic codes only. Much more work remains to be done both to replicate our results under different health delivery environments and to determine which case-detection strategy shortens outbreak detection delay and/or minimizes the burden that false alarms impose on public health professionals.

## Materials and Methods

### Ethics Statement

This study was approved by the Institutional Review Boards (IRBs) of the participating VA health systems and those of the University of Maryland, the University of Utah and the Johns Hopkins University. The risks were limited to information confidentiality involving data generated during the course of routine patient care. The study did not interfere with such care and thus did not adversely affects the rights and welfare of the participants. The IRBs granted a waiver of consent on that basis and because, given the large number of participants, the work would not have been practicable otherwise. The data collected are specified in the “Description of Procedures” section below.

### Participants

We randomly sampled outpatient clinical encounters from October 1, 2003 through March 31, 2004 at VA Maryland (VAMHCS) and at VA Salt Lake City (VASLCHCS) Health Care systems. While we aimed at sampling as broad an outpatient visit population as possible, it was nevertheless not useful to include specialized areas of care that neither diagnose nor treat ARI, such as psychiatry or ophthalmology. We therefore limited our sampling to outpatient care areas where at least two encounters were assigned ICD-9 codes related to ARI (see below) during the study period. Individual patients were sampled only once.

### Description of Procedures and Statistical Methods

#### Reference record review

A manual review of EMR entries constituted the reference standard for ARI case detection. The unit of analysis was the calendar day of an index outpatient encounter. A trained abstractor reviewed all EMR entries during the calendar day of each index encounter for documentation indicative of ARI. Predefined ARI symptoms and signs were recorded individually on an abstraction instrument (MS Access, Microsoft Corp., Redmond WA). ARI was defined as follows: [1) Positive influenza culture/antigen; OR 2) Any two of the following, of no more than 7 days duration: a) cough; b) fever or chills or night sweats; c) pleuritic chest pain; d) myalgia; e) sore throat; f) headache] AND [3) Illness not attributable to a non-infectious etiology]. All uncovered ARI cases and a 10% random sub-sample of negative records were re-reviewed by a physician, who validated the ARI-defining elements, and who could also access any part of the EMR for documentation that would indicate that a non-infectious disease could best explain the current illness. A panel of specialists in pulmonary and infectious diseases arbitrated cases where there was disagreement. Sample size was adjusted so that we could reach ∼140 reference ARI cases at each study site, a goal estimated by the need to have sufficient power to perform a regression analysis that included our planned candidate explanatory variables (EpiInfo, Version 2.2, Centers for Disease Control and Prevention).

#### Development of ARI case-detection algorithms (CDA) that use structured EMR parameters

Practicing clinicians from the research team systematically reviewed and adjucated structured or semi-structured EMR parameters that could potentially identify patients with ARI and that would be available within 24 hours of an index encounter. The parameters chosen were: a) ICD-9 diagnostic code(s) included in the existing “respiratory” groupings from SSS of national scope (either the Centers for Disease Control and Prevention (CDC) BioSense [45] or the Department of Defense's ESSENCE [46] systems) OR the ICD-9 code for fever (780.6). Note that ICD-9 codes are assigned by VA health care providers when they complete their clinical note for an outpatient visit in EMR, and are thus rapidly available for surveillance; b) vital sign abnormalities (temperature ≥38°C, respiratory rate ≥22 breath per minute, heart rate ≥100 beats per minute); c) orders for tests (complete blood count (CBC) with or without differential cell count, influenza antigen or culture, diagnostic tests for respiratory organisms other than influenza (respiratory syncytial virus, adenovirus, legionella), streptococcal throat screen, sputum culture or Gram stain, Gram stain for other respiratory specimens, blood cultures); d) requests for diagnostic imaging (chest X-ray, chest computerized tomography, any respiratory sinus imaging); and e) new prescriptions (none similar in the last 90 days), selected from the VA national formulary and grouped into the following parameters by expert consensus: cough remedies (from VA national formulary (VANF) drug classes RE-200, -301, -302, -502, -503, -507, -508, -513, -516, or codeine), “other cold remedies” (from VANF CN-900, MS-102, NT-100, -200, -400, -900, RE-99, -501); antiemetics (from VANF GA-700), antidiarrheals (from VANF GA-400), influenza-targeting antivirals (neuraminidase inhibitors or adamantanes), and alternative groupings of antibacterials (*i*: 33 antibiotics that could be prescribed for ARI (from VANF AM-051, -052, -053, -101, -102, -103, -200, -250, -300, -650 and -900); *ii*: 11 antibiotics commonly prescribed for ARI (clarithromycin, erythromycin, azithromycin, clindamycin, amoxicillin/clavulanate, amoxicillin, penicillin V, gatifloxacin, levofloxacin, cefaclor, tetracycline and doxycycline); *iii*: the three antibiotics most commonly associated with an encounter ascribed an ICD-9 code from the ESSENCE “respiratory” grouping at both sites (amoxicillin/clavulanate, azithromycin and clarithromycin). All of the data elements required to construct the above EMR parameters in the study population were transferred from the Veterans Integrated Service Technology Architecture (VistA) hierarchical database to a Structured Query Language (SQL) relational database using the Mumps Data Extractor software (Strategic Reporting Systems Inc., Peabody, MA). Data elements were included if they were entered in the EMR within the calendar day of the index encounters. Subsequent data transformations and database queries were implemented using SQL Server 2000 (Microsoft Corp., Redmond, WA).

ARI CDAs were developed independently for each study site. For a given site, the clinician-selected EMR parameters were reduced to include only those that contributed significantly to detection of reference ARI cases using backward elimination logistic regression with 95% confidence intervals [47]. Supplemental statistics in those analyses included Akaike's Information Criterion, Wald's Chi Square Test, Likelihood Ratio Test Statistic, and Drop-In-Deviance. Explanatory parameters were explored for multicollinearity with variance inflation factors and Spearman correlations. The performance of retained CDAs was summarized with standard statistical descriptors (sensitivity, specificity, positive and negative predictive value, and an estimate of the area under the Receiver-Operating Characteristic (ROC) curve [48]). Bootstrap analysis with 95% confidence intervals was conducted to test the reliability of the most successful ARI CDAs [49]. For a given CDA, the dataset was divided by placing a random sample of 80% of the data in a first subset, and the remaining 20% in a second subset. The previous analyses for the selected case detectors were performed on the 80% subset. The statistical descriptions for the case detectors in each dataset were compared and the results were found to be similar to those for the entire dataset. The case detectors were then used to predict the ARI cases in the 20% subset. Data for each case detector was resampled 100 times to ensure consistent results. All statistical analyses and ROC estimates were performed using Splus (Version 6.1, Insightful Corp., Seattle, WA).

#### Development of ARI CDAs that use unstructured clinical text

Of the many free-text data sources within the EMR, we focused on the notes typed in by providers to document outpatient visits. Our goal was first to apply simple methods using character string matching coupled with negation detection to extract information documenting ARI symptoms. If a clinical note related to an index visit contained non-negated strings related to two or more symptoms from our ARI case definition, then the index visit was labeled as “positive” for the presence of ARI.

To develop a list of search strings, we began by mapping ARI symptoms from our case definition to the Unified Medical Language System (UMLS) using the National Library of Medicine UMLS Metathesaurus® search tool [50]. The UMLS incorporates many common source vocabularies (including ICD-9, MeSH, and SNOMED) and maps concepts to a standard vocabulary represented by concept unique identifier (CUI) codes [41], [50], [51], [52], [53], [54]. We examined all of the UMLS-supplied lexical variants and semantic types related to ARI [50] to build the final list of strings. This list included 186 synonyms, term variants, and common misspellings (Table S1).

We searched for the text strings identified above in the full text of all EMR clinical notes completed on the calendar day and related to each index encounter (76,500 notes related to 15,377 encounters). To determine if concepts identified by the text strings were affirmed or negated, for example whether a patient was “coughing” or *denied* “coughing”, we used the publicly available NegEx version 2 algorithm [55]. We sought to improve the performance of the native algorithm by iteratively reviewing 10-15% of notes associated with false negative and false positive cases, then correcting problems with negation detection. Reusable text templates and checklists, commonly imported into VA note documents, drove most of the modifications to the NegEx algorithm, which included: 1) adding pre-processing steps to remove extraneous white spaces, carriage returns and line feeds; 2) identifying special characters commonly associated with imported templates (e.g. series of ----------------, ********** to indicate section breaks) or indicating embedded data-mining software objects used to automatically retrieve vital signs or medication lists; 3) identifying headings of templates that often contained a list of non-negated ARI-related strings (e.g. “Cough Assessment”, “Clinician Instructions”, “Problems to Report to Your Doctor”, “Needed For”, “Observe For”); 4) modifying the term list used by NegEx for possible negation status (e.g. adding the term “absent”); 5) using regular expressions to identify non-textual patterns where the concept identified was either affirmed or negated by special characters or abbreviations (e.g. neg, pos, [n] cough, [p] fevers, (+/-), [n/a], &, /, or *); 6) expanding the negation window up to the next full stop or to the end of a section (indicated by: [ ], (), (n), (-), or a dot).

## Supporting Information

Table S1 ARI Concepts, Synonyms, and Concept Unique Identifier (CUI) codes.
(0.19 MB DOC)
