# Correction: Combining Free Text and Structured Electronic Medical Record Entries to Detect Acute Respiratory Infections

**DOI:** 10.1371/annotation/8a0d067d-5bf7-4e2e-b7d2-56444560d66d

**Published:** 2011-01-11

**Authors:** Sylvain DeLisle, Brett South, Jill A. Anthony, Ericka Kalp, Adi Gundlapalli, Frank C. Curriero, Greg E. Glass, Matthew Samore, Trish M. Perl

The 5th author's last name was misspelled. The correct spelling of the author's name is: Adi Gundlapalli. The correct citation is: DeLisle S, South B, Anthony JA, Kalp E, Gundlapalli A, et al. (2010) Combining Free Text and Structured Electronic Medical Record Entries to Detect Acute Respiratory Infections. PLoS ONE 5(10): e13377. doi:10.1371/journal.pone.0013377.

