# Supplementary material for: Combining Free Text and Structured Electronic Medical Record Entries to Detect Acute Respiratory Infections
Source: PLoS One. 2010 Oct 14;5(10):e13377. doi: 10.1371/journal.pone.0013377 (PMC2954790; doi:10.1371/journal.pone.0013377)
Supplement: Table S1 — ARI Concepts, Synonyms, and Concept Unique Identifier (CUI) codes. (0.19 MB DOC) [file pone.0013377.s001.doc]

**Table S1:** *ARI Concepts, Synonyms, and Concept Unique Identifier (CUI) codes.*

| **Semantic Concept** | **Synonym** | **CUI** |
| --- | --- | --- |
| chills | chill NOS | C0085593 |
| chills | chill, NOS | C0085593 |
| chills | chill; NOS | C0085593 |
| chills | chills NOS | C0085593 |
| chills | chills, NOS | C0085593 |
| chills | chills; NOS | C0085593 |
| chills | chill shaking | C0085593 |
| chills | shaking chills | C0085593 |
| chills | chill | C0085593 |
| chills | chills | C0085593 |
| chills | chilling | C0085593 |
| chills | ague | C0085593 |
| chills | rigor | C0085593 |
| chills | rigors | C0085593 |
| cough | finding of cough | C0010200 |
| cough | observation of cough | C0010200 |
| cough | cough, NOS | C0010200 |
| cough | NOS, cough | C0010200 |
| cough | cough NOS | C0010200 |
| cough | cough NOS | C0010200 |
| cough | does cough | C0010200 |
| cough | productive cough | C0010200 |
| cough | cough | C0010200 |
| cough | coughing | C0010200 |
| cough | coughs | C0010200 |
| cough | cogh | C0010200 |
| cough | cocuphing | C0010200 |
| fever | Alteration in body temperature | C0015967 |
| fever | Body temperature above normal | C0015967 |
| fever | hyperthermia AND fever | C0015967 |
| fever | has a temperature | C0015967 |
| fever | high body temperature | C0015967 |
| fever | increased body temperature | C0015967 |
| fever | body temperature, increased | C0015967 |
| fever | body temperature, increased | C0015967 |
| fever | body temperature increased | C0015967 |
| fever | body temperature; increased | C0015967 |
| fever | increased; body temperature | C0015967 |
| fever | fever, NOS | C0015967 |
| fever | NOS, fever | C0015967 |
| fever | NOS fever | C0015967 |
| fever | fever NOS | C0015967 |
| fever | fever, unspecified | C0015967 |
| fever | fever, unspec | C0015967 |
| fever | fever unspec | C0015967 |
| fever | fever unspecified | C0015967 |
| fever | high temperature | C0015967 |
| fever | pyrexia NOS | C0015967 |
| fever | pyrexia, NOS | C0015967 |
| fever | pyrexia; NOS | C0015967 |
| fever | temperature elevated | C0015967 |
| fever | temperature elevation | C0015967 |
| fever | temperature increase | C0015967 |
| fever | temperature raised | C0015967 |
| fever | fever | C0015967 |
| fever | fevers | C0015967 |
| fever | pyrexia | C0015967 |
| fever | febrile | C0015967 |
| fever | febris | C0015967 |
| fever | temperature;high | C0015967 |
| fever | high;temperature | C0015967 |
| fever | temperature,high | C0015967 |
| fever | high,temperature | C0015967 |
| fever | hyperthermia | C0015967 |
| fever | pyrexia | C0015967 |
| fever | feverish | C0015967 |
| headache | Headache | C0018681 |
| headache | headache; | C0018681 |
| headache | pain in head NOS | C0018681 |
| headache | HA - headache | C0018681 |
| headache | Head pain cephalgia | C0018681 |
| headache | headache, NOS | C0018681 |
| headache | headache NOS | C0018681 |
| headache | NOS headache | C0018681 |
| headache | NOS, headache | C0018681 |
| headache | cranial pain | C0018681 |
| headache | cranial pains | C0018681 |
| headache | pain, cranial | C0018681 |
| headache | pain; cranial | C0018681 |
| headache | pain cranial | C0018681 |
| headache | pains, cranial | C0018681 |
| headache | pains; cranial | C0018681 |
| headache | pains cranial | C0018681 |
| headache | head ache | C0018681 |
| headache | headache cephalalgia | C0018681 |
| headache | headache, cephalalgia | C0018681 |
| headache | head pain | C0018681 |
| headache | pain head | C0018681 |
| headache | pain, head | C0018681 |
| headache | head, pain | C0018681 |
| headache | headache | C0018681 |
| headache | headaches | C0018681 |
| headache | cephalgia | C0018681 |
| headache | cephalgias | C0018681 |
| headache | cephalodynia | C0018681 |
| headache | cephalodynias | C0018681 |
| myalgia | myalgia unspecified | C0231528 |
| myalgia | myalgia unspec | C0231528 |
| myalgia | unspecified myalgia | C0231528 |
| myalgia | unspec myalgia | C0231528 |
| myalgia | muscle ache | C0231528 |
| myalgia | muscle aches | C0231528 |
| myalgia | muscle discomfort | C0231528 |
| myalgia | muscle pain | C0231528 |
| myalgia | pain muscle | C0231528 |
| myalgia | pain, muscle | C0231528 |
| myalgia | pain; muscle | C0231528 |
| myalgia | muscle, pain | C0231528 |
| myalgia | muscle; pain | C0231528 |
| myalgia | muscle pains | C0231528 |
| myalgia | pain muscles | C0231528 |
| myalgia | pain, muscles | C0231528 |
| myalgia | pain; muscles | C0231528 |
| myalgia | pain, muscle(s) | C0231528 |
| myalgia | pain; muscle(s) | C0231528 |
| myalgia | muscle pain/fibrositis | C0231528 |
| myalgia | muscle soreness | C0231528 |
| myalgia | muscular pains | C0231528 |
| myalgia | muscular pain | C0231528 |
| myalgia | body aches | C0231528 |
| myalgia | myalgia | C0231528 |
| myalgia | myalgic | C0231528 |
| myalgia | myodinia | C0231528 |
| myalgia | Myoneuralgia | C0231528 |
| myalgia | myosalgia | C0231528 |
| night sweats | sweat, night sweats | C0028081 |
| night sweats | night sweats | C0028081 |
| night sweats | night sweat | C0028081 |
| night sweats | sweating; night | C0028081 |
| night sweats | sweating night | C0028081 |
| night sweats | sweating, night | C0028081 |
| night sweats | night sweating | C0028081 |
| night sweats | night sweats | C0028081 |
| night sweats | nite sweat | C0028081 |
| night sweats | nite sweats | C0028081 |
| night sweats | sweat | C0028081 |
| night sweats | sweats | C0028081 |
| pleuritic chest pain | pleuristic chest pain | C0008031 |
| pleuritic chest pain | pleuristic chest pain | C0008031 |
| pleuritic chest pain | pleuristic | C0008031 |
| pleuritic chest pain | pleuritic | C0008031 |
| pleuritic chest pain | pleuritic chest pain | C0008031 |
| pleuritic chest pain | pleuritic pain | C0008031 |
| pleuritic chest pain | thoracic pain | C0008031 |
| pleuritic chest pain | pain thoracic | C0008031 |
| pleuritic chest pain | thorax pain | C0008031 |
| pleuritic chest pain | pain; thorax | C0008031 |
| pleuritic chest pain | thorax; pain | C0008031 |
| pleuritic chest pain | pain, thorax | C0008031 |
| pleuritic chest pain | thorax, pain | C0008031 |
| pleuritic chest pain | pleuristic | C0008031 |
| sorethroat | Sore Throat | C0242429 |
| sorethroat | pain in the pharynx | C0242429 |
| sorethroat | sore throat, NOS | C0242429 |
| sorethroat | sore throat NOS | C0242429 |
| sorethroat | NOS, sore throat | C0242429 |
| sorethroat | NOS sore throat | C0242429 |
| sorethroat | sore throat NOS; | C0242429 |
| sorethroat | NOS, sore throat | C0242429 |
| sorethroat | NOS sore throat | C0242429 |
| sorethroat | Pain in throat | C0242429 |
| sorethroat | sore throat | C0242429 |
| sorethroat | sore throats | C0242429 |
| sorethroat | throat, sore | C0242429 |
| sorethroat | throat sore | C0242429 |
| sorethroat | throat discomfort | C0242429 |
| sorethroat | throat pain | C0242429 |
| sorethroat | throat, pain | C0242429 |
| sorethroat | throat; pain | C0242429 |
| sorethroat | pain throat | C0242429 |
| sorethroat | pain, throat | C0242429 |
| sorethroat | pain; throat | C0242429 |
| sorethroat | pain pharynx | C0242429 |
| sorethroat | pain; pharynx | C0242429 |
| sorethroat | pharynx; pain | C0242429 |
| sorethroat | pain, pharynx | C0242429 |
| sorethroat | pharynx, pain | C0242429 |
| sorethroat | pharyngeal pain | C0242429 |
| sorethroat | pharynx discomfort | C0242429 |
| sorethroat | throat discomfort | C0242429 |
| sorethroat | throat soreness | C0242429 |
| sorethroat | sore throt | C0242429 |
| sorethroat | scratchy throat | C0242429 |
| sorethroat | scratchy throt | C0242429 |
| sorethroat | sorethroat | C0242429 |
| sorethroat | S/T | C0242429 |
